# Importance of modelling hERG binding in predicting drug-induced action potential prolongations for drug safety assessment

**DOI:** 10.3389/fphar.2023.1110555

**Published:** 2023-03-20

**Authors:** Hui Jia Farm, Michael Clerx, Fergus Cooper, Liudmila Polonchuk, Ken Wang, David J. Gavaghan, Chon Lok Lei

**Affiliations:** ^1^ Department of Computer Science, University of Oxford, Oxford, United Kingdom; ^2^ Centre for Mathematical Medicine and Biology, School of Mathematical Sciences, University of Nottingham, Nottingham, United Kingdom; ^3^ Doctoral Training Centre, University of Oxford, Oxford, United Kingdom; ^4^ Roche Pharma Research and Early Development, Pharmaceutical Sciences, Roche Innovation Center Basel, F. Hoffmann-La Roche Ltd., Basel, Switzerland; ^5^ Institute of Translational Medicine, Faculty of Health Sciences, University of Macau, Macau, China; ^6^ Department of Biomedical Sciences, Faculty of Health Sciences, University of Macau, Macau, China

**Keywords:** drug binding, drug trapping, IC50 (50% inhibition concentration), mathematical modelling, hERG channel, action potential predictions

## Abstract

Reduction of the rapid delayed rectifier potassium current (*I*_Kr_) *via* drug binding to the human Ether-à-go-go-Related Gene (hERG) channel is a well recognised mechanism that can contribute to an increased risk of Torsades de Pointes. Mathematical models have been created to replicate the effects of channel blockers, such as reducing the ionic conductance of the channel. Here, we study the impact of including state-dependent drug binding in a mathematical model of hERG when translating hERG inhibition to action potential changes. We show that the difference in action potential predictions when modelling drug binding of hERG using a state-dependent model versus a conductance scaling model depends not only on the properties of the drug and whether the experiment achieves steady state, but also on the experimental protocols. Furthermore, through exploring the model parameter space, we demonstrate that the state-dependent model and the conductance scaling model generally predict different action potential prolongations and are not interchangeable, while at high binding and unbinding rates, the conductance scaling model tends to predict shorter action potential prolongations. Finally, we observe that the difference in simulated action potentials between the models is determined by the binding and unbinding rate, rather than the trapping mechanism. This study demonstrates the importance of modelling drug binding and highlights the need for improved understanding of drug trapping which can have implications for the uses in drug safety assessment.

## 1 Introduction

The human Ether-à-go-go-Related Gene (hERG) encodes the pore-forming alpha subunit of the ion channel K_V_11.1 that conducts the rapid delayed rectifier potassium current, *I*_Kr_ (Sanguinetti et al., 1995). Reduction of *I*_Kr_ can lengthen the action potential (AP) and is associated with increased risk of arrhythmias, including Torsades de Pointes (Curran et al., 1995; Heist and Ruskin, 2010). The hERG channel is highly susceptible to block or functional inhibition by a variety of drugs (Thomas et al., 2004; Mitcheson, 2008; Vandenberg et al., 2012). This is reflected in the International Council for Harmonisation of Technical Requirements for Pharmaceuticals for Human Use (ICH) guidelines where the degree of *I*_Kr_ inhibition is part of the measures used in proarrhythmic risk assessment (Anonymous, ICH S7B, 2005), which has recently been updated to consider *in silico* simulations and pluripotent stem cell-derived cardiac myocytes experiments for integrative risk assessment (Anonymous, ICH E14/S7B Q&A, 2022).

The principal binding site of the hERG channel lies within its inner cavity (Mitcheson, 2008) and the majority of hERG-blocking drugs bind when the channel is open (Vandenberg et al., 2012). When the channel closes, some drugs can remain bound, *trapped* within the central cavity, so that unbinding can only happen on a subsequent re-opening (Mitcheson et al., 2000; Stork et al., 2007). For example, dofetilide has been shown to remain bound when the hERG channel closes (Kamiya et al., 2006), while verapamil can still unbind from the hERG channel after repolarisation (Zhang et al., 1999). Simulation studies have suggested that such *trapped drugs* may be more prone to cause arrhythmia (Di Veroli et al., 2014; Pearlstein et al., 2016).

Trapping can be investigated experimentally by studying the rate of recovery from drug block at the resting potential (Stork et al., 2007; Windisch et al., 2011). In voltage-clamp experiments using “Milnes’ protocol” (Milnes et al., 2010), the property of drug trapping can be seen as a lack of recovery from drug block during long intervals between successive channel-opening pulses: Non-trapped drugs can dissociate during these intervals so that current amplitude is restored on the next opening, while for trapped drugs the current remains diminished upon re-opening.

Drug effects can be incorporated into mathematical models of ion currents, which can then be embedded in cell models to study their effect on the action potential duration (APD) (see, e.g., Mirams et al., 2011). The predicted APD changes can then be used directly, as a surrogate for changes in the QT interval, or in multi-scale models that go up to tissue, organ, or even ECG level (Brennan et al., 2009; Mirams et al., 2012). A straightforward way of including drug effects in ion current models, is to assume that a certain fraction of a cell’s channels are blocked, and to scale the current’s *maximum conductance* (or permeability) variable accordingly (see, e.g., Davies et al., 2011). Alternatively, drug effects can be modelled by changing channel transition rates (Tsujimae et al., 2007) or by adding new states to a multi-state channel model (Di Veroli et al., 2012). This strategy of adding states has also been used to study trapping (Di Veroli et al., 2014; Li et al., 2017).

**FIGURE 1:**
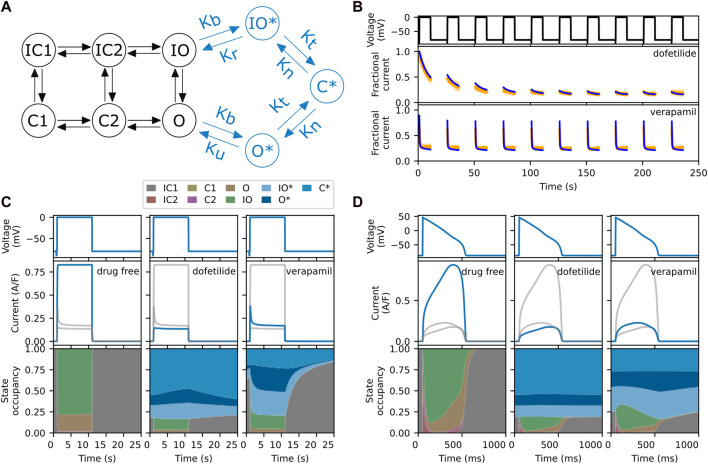
**(A)** The six-state model by Li et al. (2016) (in black) and its extension to model drug block and trapping by Li et al. (2017) (black and blue states). **(B)** Fractional block predictions for 10 paces of Milnes’ protocol (top) for the SD model with a trapped drug, dofetilide (middle) and a non-trapped drug, verapamil (lower). The SD model’s response (blue) is overlaid on the experimental data (orange) from Li et al. (2017). **(C)** Steady state response (after 1,000 paces) for the SD model during a single step of Milnes’ protocol, for a drug-free (left), trapped (middle), and non-trapped configuration (right). The lower row shows the state occupancy: the fraction of channels in any one state at a given time. **(D)** Like panel C, but using a predetermined elongated AP signal (1 s) instead of a rectangular pulse (25 s). For comparison, the grey lines in each column of panels C and D show the data from the other columns. Note that the labelling of the IC and C states, adapted from the original model, does not always correspond to their respective physical states.

Here, we will focus on two particular models of hERG block. The first, shown in black in Figures 1A is the six-state model by Li et al. (2016). In this model, drug block can be simulated by scaling the conductance variable, and we shall refer to it as the *conductance scaling* (CS) model. The second model, by Li et al. (2017), extends the first with the three additional states, shown in blue in Figure 1A. These additional states are used to model drug block and trapping, and we shall refer to the extended nine-state model as the *state dependent* (SD) model. Both models are described in detail in Section 2.

An interesting feature of the SD model is that its transition rates can be adjusted to mimic the effects of different drugs. This is illustrated in Figure 1B, where we show the experimental data (orange) and model’s response (blue) to Milnes’ protocol (top panel) when parameterised for dofetilide, a trapped drug (middle panel) and verapamil, a non-trapped drug (lower panel) for hERG (Li et al., 2017). The drug concentrations of dofetilide and verapamil were 30 nM and 1 μM, respectively, giving 75%–90% steady state block of hERG. In both cases, *I*_Kr_ rapidly activates during the high-potential pulses, but its magnitude then diminishes as the drug binds to and blocks the open channels. In the trapped case, the drug stays bound during the interpulse interval, so that the current at the start of each pulse maintains the diminished magnitude of the previous pulse, and this process continues until the drug binding saturates. In the non-trapped case, the drug dissociates during the interpulse interval, so that each successive pulse shows a similar *I*_Kr_ response. This mechanism is illustrated further in Figure 1C, which shows the state occupancy during a single pulse of Milnes’ protocol. With high trapping tendency, the channel stays in a drug-bound (blues) states during the interpulse interval, while with low trapping tendency the channel rapidly returns to a closed or inactivated state. Figure 1D shows the response and state occupancies when a predetermined elongated AP signal is applied. In this case, both drugs remain bound between pulses (APs). Since the trapping tendency of the drugs is not strongly reflected within the state occupancy under an AP-clamp, it raises the question of whether we need to include these complex mechanisms when simulating the effect at the AP level.

For certain compounds, the SD model allows us to investigate complex binding/unbinding mechanisms and how such drug-channel interactions contribute to changes in the AP. The simpler CS model, which does not capture drug-channel interactions, is often thought to oversimplify drug effects on ion channels, thus providing inaccurate predictions of a drug’s arrhythmogenic potential. However, inclusion of complex drug-binding features significantly increases the number of model parameters and enhances the challenge of accurately parameterising the model (Whittaker et al., 2020).

In this study, we compare the complex SD model with the simpler CS model, and the conditions under which these models are similar. In particular, we compare and assess the differences of either model, as judged from the predicted effects on the AP, APD and qNet. As in the example above, we will look at two SD model parameterisations representing “synthetic drugs” with properties similar to dofetilide (high trapping tendency) and verapamil (low trapping tendency), and we compare model predictions under different protocols. We further identify drug properties (SD model parameterisations) where AP effects of the SD and CS models are indistinguishable.

## 2 Materials and methods

We first describe the hERG channel models used in this study, then the procedure used to make the hERG channel models comparable. Next, we describe the AP model, synthetic drugs, and protocols used to compare the hERG channel models. The hERG channel models are included as part of the AP models for comparing their effects on APs. Finally, we explain the details of the sensitivity analysis performed on the state-dependent drug block (SD) model and the metrics used to quantify the difference in the APs.

As an overview, we take the SD model, use it as a reference to calibrate the ionic conductance of the conductance scaling drug block (CS) model, then input these hERG channel models into an AP model for AP comparison.

### 2.1 hERG channel base-model

The hERG channel base-model used in this study is the model by Li et al. (2016), as shown in Figure 1A in black. It is a six-state Markov model with two inactivated closed (IC) states, two closed (C) states, an inactivated open (IO) state, and an open (O) state. Scaling the ionic conductance of the hERG channel base-model, which is equivalent to inhibiting the hERG current, gives the conductance scaling drug block (CS) model. Extension with drug bound states of open bound (O*) state, inactivated open bound (IO*) state, and closed bound (C*) state, to the hERG channel base-model gives the SD model, as shown in Figure 1A (both black and blue components).

### 2.2 State-dependent drug block model of hERG

The SD model is based on the model by Li et al. (2017). The transition rate parameters for the hERG channel base-model are taken from Li et al. (2016), Li et al. (2017). The pharmacodynamic component is described by the drug bound states of open bound (O*) state, inactivated open bound (IO*) state and closed bound (C*) state, and their respective transition rates as shown in Figure 1A.

In this model, compounds can bind to the O or IO states of the hERG channel and transition to the O* or IO* states, respectively, with a binding rate (*K*_*b*_) given by

$$K_{b}=K_{u}\times E_{\text{max}}\left(D\right),$$ (1)

$$=K_{u}\times K_{\text{max}}\times \frac{D^{n}}{D^{n}+{EC}_{50}^{n}},$$ (2)

 Where *K*_*u*_ is the unbinding rate and *E*_max_ is a sigmoid function describing a drug’s response at concentration *D*. The function is scaled by *K*_max_, the maximum response of a drug when it is at its saturating concentration. Finally, *n* is the Hill coefficient and EC_50_ is the drug concentration when the binding rate is at 50% of its maximum. The compound can be “trapped” in the C* state with a trapping rate (*K*_*t*_) that is fixed at *K*_*t*_ = 3.5 × 10^−5^ ms^−1^ (Li et al., 2017). The rate at which the drugs get “untrapped” (*K*_*n*_) is defined as

$$K_{n}=K_{t}\times X\left(V\right),$$ (3)

$$=K_{t}\times \frac{1}{1+e^{-\left(V-V_{\text{half}-\text{trap}}\right)/6.789}},$$ (4)

 where *V*_half−trap_ is the membrane voltage when *K*_*n*_ is half the trapping rate *K*_*t*_. According to Li et al. (2017), the function *X*(*V*) is adapted from the hERG channel’s steady state activation of the O’Hara et al. (2011) model. A trapped drug can be modelled with either a high trapping rate (*K*_*t*_) or a low untrapping rate (*K*_*n*_), and in this case *V*_half−trap_ is the only parameter that controls the ratio of trapping to untrapping rate. Finally, the transition rate from the IO* state to the IO state, *K*_*r*_, is defined to maintain the microscopic reversibility of the system.

### 2.3 Calibration of a simple conductance block model

To reveal how the SD model alters *I*_Kr_ and the AP, we compared the SD model with a simple instantaneous full-state drug block model, the CS model. To ensure that the SD model and the CS model are comparable, we use simulated data from the SD model to calculate the appropriate scaling of the CS model to ensure that the peak *I*_Kr_ of both models is equal, as detailed below.

The CS model is equivalent to modelling the current inhibition by scaling the ionic conductance thereby reducing the overall flow of ions across the channel. This model assumes that the half maximal inhibitory concentration (IC_50_) of a drug on the hERG channel does not change with the experimental protocols. We note that the IC_50_ of a drug can be protocol-dependent as demonstrated in the later sections, and we acknowledge the differences in the results. However, investigation into such a dependence is not the aim of this study, therefore we use Milnes’ protocol (Milnes et al., 2010, see Section 2.6) to simulate the models as in Li et al. (2017).

**FIGURE 2:**
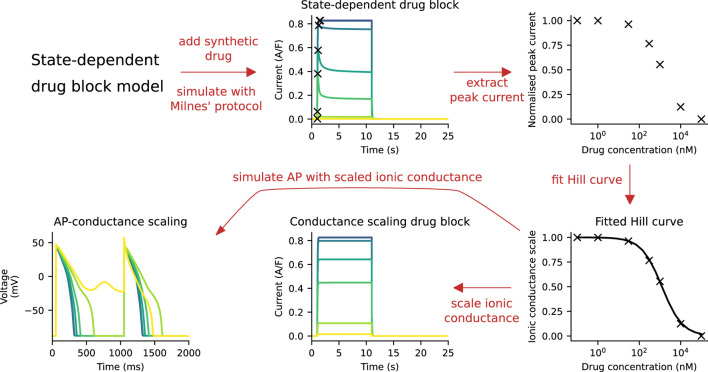
A schematic of the process of fitting the CS model to the SD model. First, simulations are run with the SD model and Milnes’ protocol at various drug concentrations. The peak of the hERG currents are extracted and normalised, and a Hill curve is fitted to the normalised data points. This Hill curve is then used to scale the ionic conductance of the CS model. Finally, the simulated APs of both models are compared.

The ionic conductance of the hERG channel was scaled to capture the drug response, such that the peak current of the CS model under Milnes’ protocol matched that of the SD model. A schematic of the fitting procedure is shown in Figure 2. We assumed that the SD model captures the experimental data presented in Li et al. (2017), which can be used as a reference point. For each “synthetic drug” (see Section 2.5), the SD model *I*_Kr_ was simulated at a range of drug concentrations using Milnes’ protocol. The peaks of these *I*_Kr_ were normalised and then fitted to the Hill curve. The Hill curve parameters—the Hill coefficient and IC_50_—were determined by minimising the mean squared error between the peak currents and the Hill curve. The ionic conductance of the CS model was then scaled with the drug response output obtained from the Hill curve function. The peak hERG current of the CS model will then have the same peak current as the SD model for a given drug and concentration, as required.

The optimisations of the Hill curves to the peak of hERG currents were performed with the covariance matrix adaptation-evolution strategy (CMA-ES) algorithm (Hansen et al., 2003) in the PINTS software (Clerx et al., 2019).

### 2.4 AP model

To study the effect of the hERG channel model at the level of APs, the AP model by Dutta et al. (2017) was used. The model was based on the Li et al. (2017) model, and to allow better quantification of each individual current on the AP, the ionic conductances of five currents were scaled (Dutta et al., 2017), including *I*_Kr_, the slow rectifier potassium current (*I*_Ks_), the inwardly rectifying potassium current (*I*_K1_), the L-type calcium current (*I*_CaL_), and the late sodium current (*I*_NaL_). The AP model, when its hERG channel component is the SD model, is referred to as the AP-SD model; the AP model with its hERG channel component replaced with the CS model is referred to as the AP-CS model.

### 2.5 Synthetic drugs

The binding dynamics on the hERG channel of all of the twelve drugs in Li et al. (2017) were taken as the synthetic drugs in this study; the parameters of the 12 synthetic drugs are provided in the Supplementary Material. Here, dofetilide and verapamil were chosen as examples to compare trapping and non-trapping properties of the hERG channel, respectively. Although verapamil is a multi-ion channel blocker, we considered only its effect on the hERG channel, so any multi-channel effects on the AP were deliberately neglected, reflecting the focus of this study being only on the hERG channel binding effects on the AP. The synthetic drug with parameters describing dofetilide is referred to as *example drug T*, while the synthetic drug with parameters describing verapamil is referred to as *example drug N*. Only these two drugs out of the 12 synthetic drugs tested are shown in the main results, and the results of the remaining synthetic drugs are provided in the Supplementary Material.

### 2.6 Protocols

Four voltage protocols were used in this work (Figure 6A). Firstly, Milnes’ protocol as modified by Li et al. (2017) from that of Milnes et al. (2010). This modified Milnes’ protocol aims to identify trapped and non-trapped drugs. Experimental data obtained from the stimulation with this protocol was used to fit the parameters of the SD model. Therefore, this modified Milnes’ protocol was used as a reference for the model comparison.

The remaining voltage protocols, *Pneg80*, *P0*, and *P40*, were taken from Gomis-Tena et al. (2020). These protocols were designed so that the channel’s state occupancy of a certain state is maximised. When the hERG channel model is stimulated by the Pneg80, P0, and P40 protocols, the channel will most likely be in the closed, open, and inactivated states, respectively, as discussed in Gomis-Tena et al. (2020). Details of all protocols are given in the Supplementary Material.

### 2.7 Sensitivity analysis

The behaviour of the drug-related component of the SD model is governed by its transition rates, which in turn are defined by the five drug-dependent parameters. To understand the role of each drug-related parameter in the SD model, we performed a sensitivity analysis on the APD_90_ difference between the AP-SD and the AP-CS model.

First, we simplified the SD model by normalising the drug concentration *D* input to the EC_50_ value. The normalised drug concentration is therefore defined as

$$\tilde{D}=\frac{D}{{EC}_{50}}.$$ (5)

 This reduces the number of parameters to four.

Second, we observed that the Hill coefficient *n* affects only the range of drug concentration where AP prolongation and early after depolarisation (EAD)-like behaviour are observed. To confirm this, we varied the value of *n* for each synthetic drug and monitored the APD_90_ differences between the two models. The APD_90_ differences were quantified by the root mean square difference (see Section 2.8). The root mean square difference (RMSD) of APDs fluctuated within a range of only 25 ms as *n* was varied (see Supplementary Material). The model was thus further simplified to three parameters, namely, the *V*_half−trap_, *K*_max_, and *K*_*u*_. We then performed a detailed sensitivity analysis on this 3-dimensional space.

In the sensitivity analysis, we repeated the model comparison procedure as shown in Figure 2 for a range of parameter values in the simplified SD model. The three parameters of the simplified SD model describe a hypothetical drug which is termed a “virtual drug”. Sweeping through the parameter space effectively performed a virtual drug screening for all possible drug binding kinetics of hERG, within the SD model.

The two models, the AP-SD model and the AP-CS model, were considered to be similar if the RMSD of the APD_90_ values are small. The parameter space around the boundary surface, where the RMSD values between the two models are less than 30 ms, were sampled more densely to obtain higher resolution.

### 2.8 Metrics for APD_90_ difference

The RMSD and the mean difference (MD) between the APD_90_ of the AP-SD and the AP-CS models were used to quantify the model comparison. The RMSD measures the magnitude of the APD_90_ difference between the models, and is given by

$$RMSD=\sqrt{\frac{\sum_{i=1}^{N}{\left({\mathrm{SD}}_{i}-CS_{i}\right)}^{2}}{N}},$$ (6)

 where *N* is the total number of data points, excluding data points where EAD-like behaviours are observed, and *SD* and *CS* are the APD_90_ values of the AP-SD model and the AP-CS model, respectively.

The MD measures the actual difference between the two models,

$$MD=\frac{\sum_{i=1}^{N}\left({\mathrm{SD}}_{i}-CS_{i}\right)}{N}.$$ (7)

 A positive value of MD implies that the sum of the APD_90_ values of the AP-SD model are higher than the AP-CS model, while a negative value implies the opposite.

The signed RMSD was used to indicate the magnitude and direction of the APD_90_ difference between the AP-SD model and the AP-CS model:

$$RMSD_{\text{signed}}=RMSD\times \frac{MD}{\left|MD\right|}.$$ (8)

### 2.9 Simulations

All simulations, including voltage-clamp and current-clamp experiments, were run with Python 3.8 using Myokit 1.33 (Clerx et al., 2016) with the CVODE solver (Hindmarsh et al., 2005). The absolute tolerance and relative tolerance were set to 10^−7^ and 10^−8^, respectively.

## 3 Results

### 3.1 CS model replicates SD model at steady state for a trapped drug example

Figures 1B, C show that the SD model can capture the trapping properties of a drug, observed in the lack of recovery from block and the state occupancy of drug bound states. However, it is not clear whether a simpler AP-CS model could replicate the AP prolongation by the AP-SD model that has state dependency and a trapping mechanism included. We systematically compared the APs of the two models for the example drug T and the example drug N using the methods described in Section 2.3; Figure 2.

**FIGURE 3:**
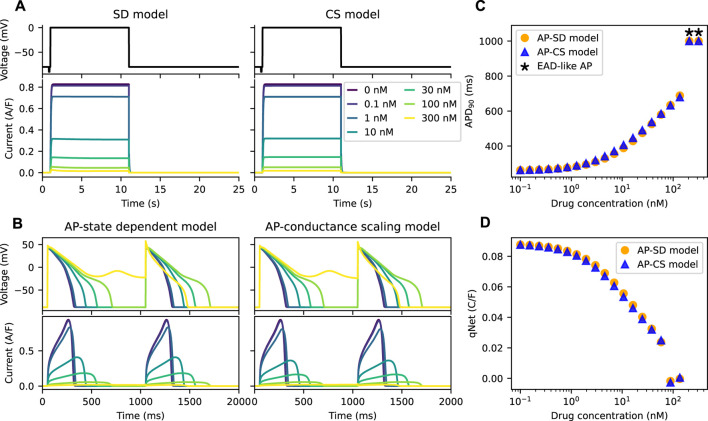
The AP-CS model can provide a reasonable approximation of the AP-SD model for an example drug T. **(A)** The matching of the peak hERG currents simulated by both drug block models under Milnes’ protocol (top row) at various drug concentrations of an example drug T. **(B)** The AP (first row) and its hERG current (second row) for the AP-SD model and the AP-CS model at steady state. **(C)** The APD_90_ values of the AP-SD model and the AP-CS model under the effect of example drug T. APs that show EAD-like behaviour are indicated with an asterisk. **(D)** The qNet values of the AP-SD model and the AP-CS model under the effect of example drug T. qNet values of APs with EAD-like behaviours are not shown.

Figure 3A shows the *I*_Kr_ of both the SD model and the CS model with an example drug T present there throughout under Milnes’ protocol. By design, the peaks of the *I*_Kr_ of the two models are the same at various drug concentrations of the example drug T, which give the same dose-response effect. The APs of the two models (the AP-SD model and the AP-CS model) with the example drug T are given in Figure 3B, showing two pulses of the AP at steady state, together with their corresponding *I*_Kr_. As the drug concentration increases, the amplitude of the *I*_Kr_ decreases, and the APD increases. The two models show similar AP behaviours, although the AP-SD model simulated *I*_Kr_ at 10 nM of example drug T has a bigger amplitude and a shorter period when the *I*_Kr_ is positive. The differences give rise to a slightly shorter APD because *I*_Kr_ aids in the repolarisation of APs. Moreover, both models show an EAD-like behaviour at the same drug concentration (300 nM) of the example drug T.

In Figure 3C, the APD_90_ values of APs simulated from the AP-SD model and the AP-CS model for an example drug T were calculated. APs that show EAD-like behaviours are indicated with an asterisk. The APD_90_ values of both models are similar, consistent with the APs shown in Figure 3B. Additionally, the qNet values, a proarrhythmic risk marker, of example drug T shown in Figure 3D are similar for both models. Therefore the CS model can replicate the SD model for an example drug T at steady state.

**FIGURE 4:**
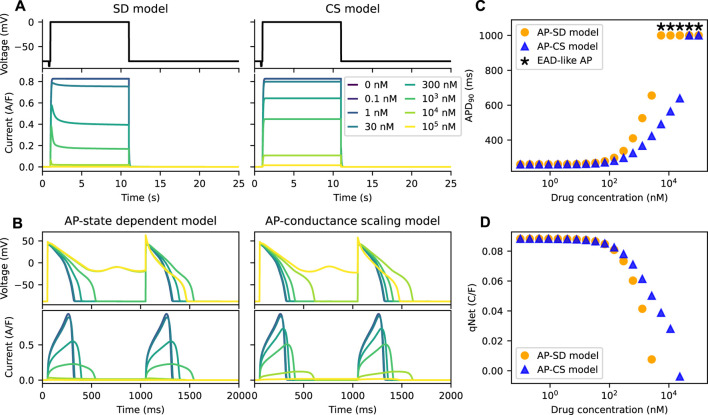
The AP-CS model is less successful at replicating the model behaviour of the AP-SD model for an example drug N. **(A)** The comparison of the *I*_Kr_ simulated by both the SD model and the CS model after calibration of the hERG ionic conductance for an example drug N. The top row shows Milnes’ protocol used to stimulate the hERG channel models. **(B)** The AP (first row) and its hERG current (second row) for the AP-SD model and the AP-CS model at steady state. **(C)** The APD_90_s of the AP-SD model and the AP-CS model under the effect of an example drug N. APs that show EAD-like behaviour are indicated with an asterisk. **(D)** The qNet values of the AP-SD model and the AP-CS model under the effect of example drug N. qNet values of APs with EAD-like behaviours are not shown.

We further compared the APs of the models for an example drug N, a “non-trapped” drug; the non-trapping phenotype of the drug is captured by the small value of *V*_half−trap_. Figure 4A shows the *I*_Kr_ of the two models under the effect of the example drug N. The *I*_Kr_ simulated by the SD model show dips after the initial increase at drug concentrations higher than or equal to ∼300 nM. Since the peak current is used to define the inhibition level of a drug, which is commonly done for IC_50_ calculations, the total amount of *I*_Kr_ simulated by the CS model is higher than the SD model.

Figures 4B, C compare the APs and their corresponding *I*_Kr_ of the AP-SD model and the AP-CS model for the example drug N. The *I*_Kr_ and the APs are significantly different between the two models. At high drug concentrations the AP-SD model shows a longer APD and a lower *I*_Kr_ amplitude than the AP-CS model. The AP of the AP-SD model is prolonged more than the AP-CS model and displays EAD-like behaviours at lower drug concentration. The example drug N, for which the untrapping rate is higher and the binding rate is lower than the example drug T, generated lower APD_90_s with the AP-CS model. Similarly, the qNet values of example drug N (Figure 4D) are different between the two AP models. Example drug T and example drug N are taken as examples to show the difference in APD_90_ and qNet for drugs with varying trapping tendency. The same analysis was repeated for other drug compounds (see Supplementary Material).

### 3.2 Trapping properties are apparent in transient phase

**FIGURE 5:**
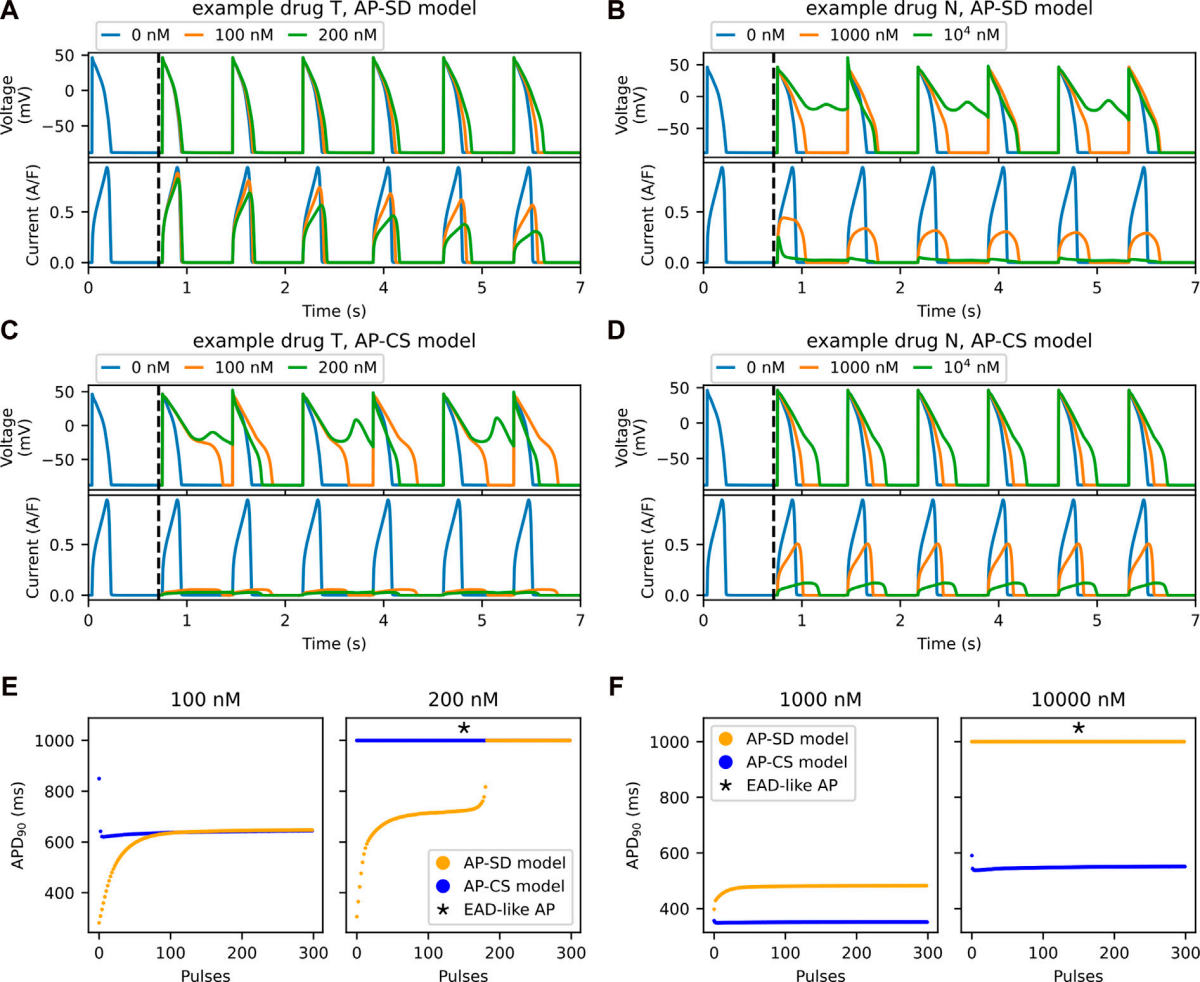
The accumulation of drug compounds at the channel due to being trapped is well-observed in the transient phase. Example drug T shows a longer transient phase than an example drug N in the SD model. The first six pulses of AP (top row) and its hERG current (bottom row) for the AP-SD model after the addition of **(A)** an example drug T and **(B)** an example drug N. The vertical black dashed lines indicate the addition of drug into the system after 1,000 pulses of pacing. The first six pulses of the AP (top row) and the hERG current (bottom row) for the AP-CS model after the addition of **(C)** example drug T and **(D)** example drug N, of which its hERG ionic conductance is scaled to model the drug effect. **(E)** The APD_90_ values of both the AP-SD model and the AP-CS model are compared at 100 and 200 nM of example drug T. **(F)** The APD_90_ values of both the models are compared at 1,000 nM and 10^4^ nM of example drug N.

The main difference between the two drug block models is the inclusion of the trapping mechanism in the hERG channel model. The feature of this mechanism is the accumulation of the drug compound in the channel due to “trapping”. Figures 5A–D show the progression of the APs and their *I*_Kr_ for the example drug T and the example drug N with the two AP models. In Figure 5A, the *I*_Kr_ decreases gradually with time for the AP-SD model with the example drug T, and the AP prolongation increases. In contrast, the *I*_Kr_ and the APs for the example drug N in the AP-SD model are relatively stable, as shown in Figure 5B. The AP-SD model displays a shorter transient phase with the example drug N than with the example drug T, which is due to the differences in the trapping phenotype of these synthetic drugs. Figures 5C, D compare the AP-CS model with the example drug T and the example drug N, respectively, and show that the APs and the *I*_Kr_ do not change much between pulses.

The APD_90_ values from Figures 5A–D are given in Figures 5E, F for the example drug T and the example drug N, respectively. For the example drug T (Figure 5E), the AP-SD model showed a longer transient phase, then stabilised to generate the same APD_90_ as the AP-CS model. By contrast, the two models with the example drug N (Figure 5F) had a short transient phase with different APD_90_ values at steady state.

### 3.3 AP prolongation is dependent on the protocol used to stimulate the SD model

**FIGURE 6:**
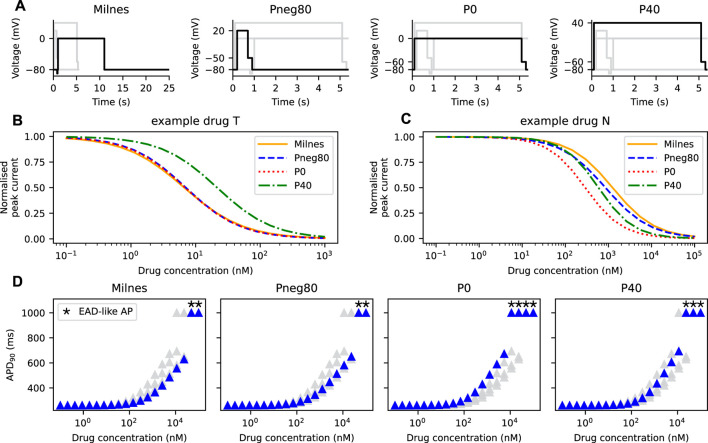
The Hill curves for an example drug T and an example drug N are protocol dependent, thus affecting the resultant APD prolongation. **(A)** The four protocols used to calibrate the hERG ionic conductance from the SD model. Note the differences in the time axes for visualisation purposes. The Hill curves of the peak *I*_Kr_ simulated from the SD model under the protocols given in panel A for **(B)** an example drug T and **(C)** an example drug N. **(D)** The APD_90_s of the AP-CS model under the effect of an example drug N with its hERG conductance scaled based on the Hill curves shown in panel C.

In all model comparisons shown in Figures 3, 4, we have assumed that the IC_50_ of a drug is independent of the protocol used to measure its response. Thus, the IC_50_ can be used to replicate drugs effect in any drug block models (Mirams et al., 2011). However, the assumption does not always hold (Kirsch et al., 2004; Yao et al., 2005). Here, we demonstrate the change in the APD_90_ when different protocols are used to generate the *I*_Kr_ for drug characterisation. Figure 6A shows Milnes’, *Pneg80*, *P0*, and *P40* protocols that are used to stimulate the SD model. The normalised peak of the *I*_Kr_ simulated with each of the given protocols for both the example drug T and the example drug N are shown in Figures 6B, C, respectively. The example drug T (Figure 6B) yields similar dose-response curves for all of the protocols, except for the P40 protocol, while the example drug N (Figure 6C) displayed different dose-response curves for all protocols. Using the Hill curves in Figure 6C to characterise the drug effect for the CS model (see Section 2.3; Figure 2), the APs of the AP-CS model were simulated and quantified. Figure 6D shows the APD_90_s of the simulated APs with an example drug N. While using the Pneg80 protocol gave similar APD_90_s to using Milnes’ protocol, the use of P0 and P40 protocols generate higher APD_90_ values, causing EAD-like behaviours to appear at lower concentrations of the example drug N. Example drug T and example drug N are taken as examples. The Hill curves for all the protocols with the other drug compounds are given in the Supplementary Material.

### 3.4 Drugs were approximated to have lower steady state APD_90_ values with the AP-CS model

A sensitivity analysis was performed on the simplified SD model as described in Section 2.7, where the SD model was simplified to just three drug-related parameters—*V*_half−trap_, *K*_max_, and *K*_*u*_—without loss of generality. Given a virtual drug from the parameter space, the procedure shown in Figure 2 was repeated (Section 2.3). The APD_90_ differences between the AP-SD model and the AP-CS model were measured by the signed RMSD (Equation 8).

**FIGURE 7:**
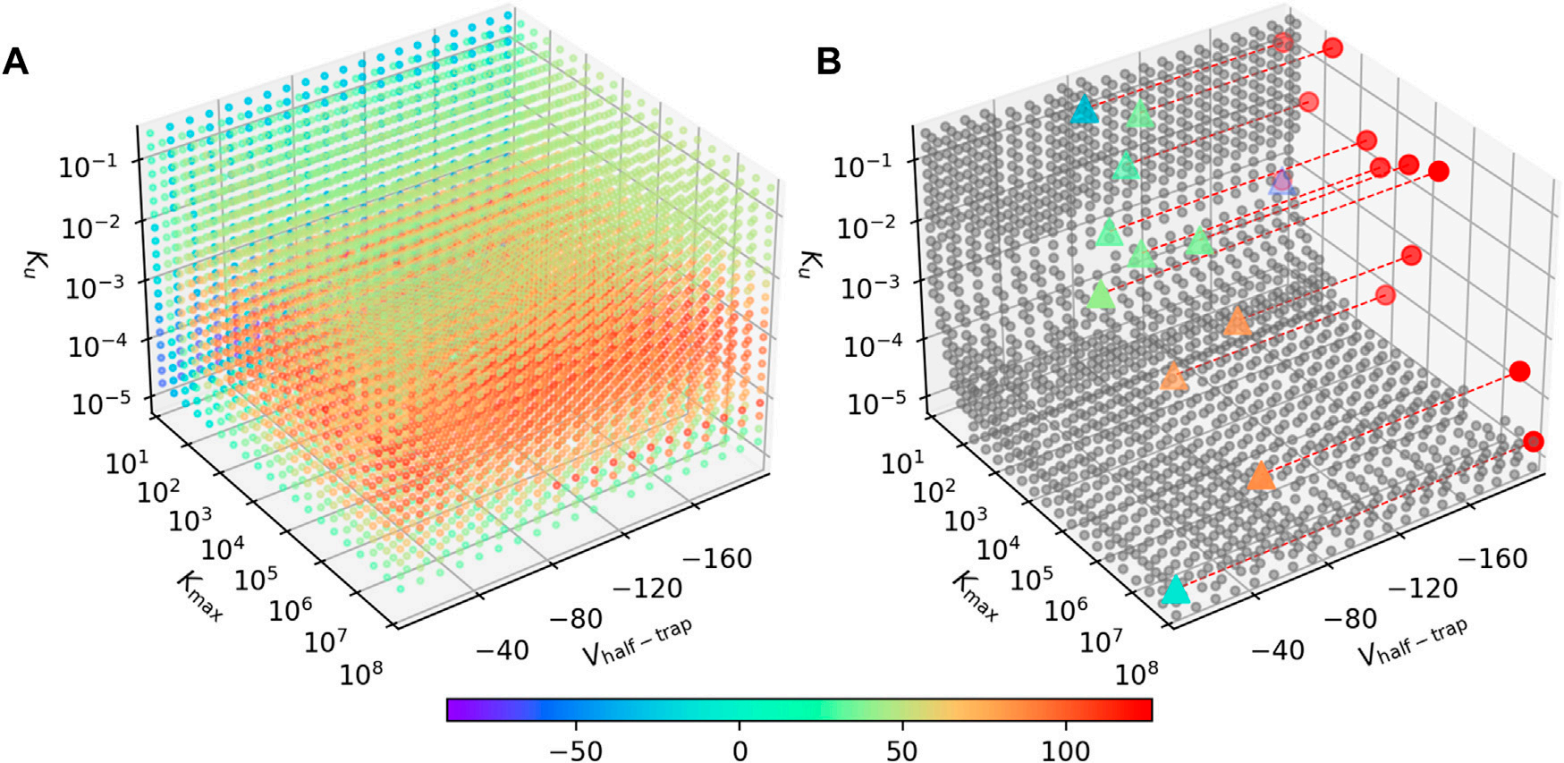
The AP-SD model is most likely to return higher APD_90_s than the AP-CS model. **(A)** The APD_90_ differences for combinations of *V*_half−trap_, *K*_max_ and *K*_*u*_ parameters. The color of the markers indicate the signed RMSD of each virtual drug in the parameter space. **(B)** The grey circles are parameter value combination where the signed RMSD is between −30 and 30 ms. The triangles are the synthetic drugs taken from Li et al. (2017), color coded with their signed RMSD value. These triangles are projected to the *K*_max_—*K*_*u*_ plane as red circles for better visualisation. Some data points are missing due to numerical issues with these parameter combinations.

Figure 7A shows the signed RMSD for combinations of the three parameters. Each point represents a virtual drug, while its color indicates the APD_90_ difference between the AP-SD model and the AP-CS model when the virtual drug is added. The majority of the virtual drugs resulted in a positive signed RMSD value, indicating that the AP-SD model generated APs that had longer durations than the AP-CS model. It is also shown that the signed RMSD shifts from positive to negative as *K*_max_ decreases. The virtual drugs are generally predicted to cause shorter AP prolongation when using the AP-CS model.

The parameter space where the two models give a similar APD_90_ value (RMSD < 30 ms) is limited, as shown in Figure 7B. For these parameters, the AP-SD model and the AP-CS model are interchangeable when we measure only the APD. The triangles show the positions of the synthetic drugs in the parameter space. Synthetic drugs that lie within the boundary surface are listed in the Supplementary Material.

The results show that *V*_half−trap_ plays a small role in affecting the differences between the AP-SD model and the AP-CS model. *K*_max_ and *K*_*u*_ are the major driving forces for the changes in the APD_90_ difference between the two models. Furthermore, at low values of *K*_max_, *K*_*u*_ does not affect the APD_90_ difference as much; for large *K*_max_, the two models differ the most around *K*_*u*_ ≈ 5 × 10^−4^. A different viewing angle of Figure 7B is available in the Supplementary Material.

## 4 Discussion

This study has compared the AP simulated by the AP-CS model and the AP-SD model. The CS model reduces the ionic conductance to approximate the effect of *I*_Kr_ reduction induced by drug binding to the hERG channel, while the SD model includes the state dependency and the trapping mechanism to replicate the drug’s effect. Our comparison intended to explore whether the more complex SD model and the simpler CS model give the same prediction of hERG inhibitor-induced APD change. A sensitivity analysis of the simplified SD model, with the *V*_half−trap_, *K*_max_ and *K*_*u*_ parameters, showed that there is only a small region of the parameter space where the two models produce similar steady state APD_90_ values (RMSD of the steady state APD_90_s < 30 ms). The AP-CS model suggested that the majority of the virtual drugs induce lower steady state APD_90_ values than the AP-SD model, leading to possible lower arrhythmic risk.

AP simulations of the AP-SD model and the AP-CS model with an example drug T, a trapped drug, show similar AP prolongations and qNet values at steady state (Figure 3). The resulting APD_90_ differences between the models contradict our expectations, as it was hypothesised that the AP-SD model with a trapping mechanism would predict trapped drugs like dofetilide to cause more AP prolongation (Pearlstein et al., 2016). It was believed that the accumulation of the drug at the channel (“trapped”) could inhibit the current more as compared to no trapping, thus prolonging the APD further. On the other hand, for the example drug N, a non-trapped drug, the AP-CS model predicted less AP prolongation than the AP-SD model (Figure 4), conforming to expectations. The difference in the qNet values for example drug N suggest that the drug could be categorised in different risk categories if the AP-SD model or the AP-CS model is used. The results suggest that the AP prolongation prediction of drugs like dofetilide does not differ between the AP-SD model and the AP-CS model.

The effects of drug trapping in the hERG channel become apparent during the transient phase as it takes more pulses to achieve steady state (Figure 5). Trapped drugs accumulate at the channel, without recovering from the block during the interpulse interval, and decrease the current pulse by pulse until steady state, creating a longer transient phase; non-trapped drugs unbind and rebind to the channel in between pulses of protocol, seemingly achieving steady state. However, for the example drug T, the AP-SD model and the AP-CS model predict similar steady state APD changes, suggesting that trapping is likely not a key driver for the differences in APD changes.

To further explore the observation, our sensitivity analysis on the drug-related parameters of the SD model demonstrated that the AP-SD model and the AP-CS model predict significantly different AP prolongation for the majority of the virtual drugs. Therefore, modelling drug effect by simply reducing the ionic conductance is most likely insufficient to capture the dynamics of drug binding, especially at the AP level. However, we also observed only a small change in the APD_90_ difference between the two models when *V*_half−trap_ changes, which implies that no extra dynamics can be observed from the addition of the *V*_half−trap_ parameter or the *trapping component*, confirming our previous observation. Furthermore, the parameters controlling the binding and unbinding rates—*K*_max_ and *K*_*u*_—determine the difference in the steady state AP prolongation predictions between the two models (Supplementary Figure S13). We note that it does not imply *V*_half−trap_ has no influence on the APD_90_ values; although the difference in the APD_90_ values are small, the APD_90_ values vary with *V*_half−trap_ as shown in Supplementary Figures S13, S14. This difference in AP prolongation for majority of the parameter space implies that one cannot assume that with the same baseline AP model, switching between the SD model and the CS model can achieve the same prediction results as the outcome of the model training. It is not possible to determine the best model in this study as it depends on what type of input data the model is trained on. Since it is common practice to acquire IC_50_ data, it is much pragmatic to use the CS model. Without re-evaluation of a drug on the SD model, it cannot be assumed that the SD model can replace the CS model, or *vice versa*. It is important that the model is used within the context of use which the model is trained and validated on (including the type of input data).

One of the limitations of this study is that we assumed that the SD model can replicate the experimental data perfectly and thus fitted the CS model to the Hill curve generated from the SD model. Moreover, the six-state Markov model and the Dutta et al. (2017) model, two well-known models, were used as the hERG base-model and AP base-model, respectively, but changing the choice of the models could affect the behaviour of the APs and hence the differences in APs between the SD model and the CS model (Clayton et al., 2020; Lei et al., 2020). Furthermore, in the sensitivity analysis, the ranges of the parameter values were determined by the minimum and maximum of each of the parameters of the synthetic drugs, and the trapping rate of the SD model was fixed in the same manner as it was done in the original model, limiting the exploration of the model dynamics. Finally, we emphasise that our focus is on the drug effect on the hERG channel only, and specifically on the effect of inclusion of the state-dependent drug binding of the hERG channel. Investigation of the effect of other ion channels is out of scope of this study. Therefore, this study is not an integrative and comprehensive assessment of the change in AP or proarrhythmic risk of the drug compounds, particularly for a multi-channel blocker such as verapamil.

In this study, we have shown that the APD_90_ difference between the AP-SD model and the AP-CS model for the example drug T and the example drug N is not consistent with their trapping phenotypes, which are differentiable only in the transient phase. The ratio of trapping to untrapping rate, defined in the SD model with *V*_half−trap_, does not affect the APD_90_ differences between the two models. The binding rates and unbinding rates, on the other hand, are the main determinants of the APD_90_ differences. This study demonstrates the importance of modelling drug binding and highlights the need for improved understanding of drug trapping which can have implications for the uses in drug safety assessment.

## Data Availability

The datasets presented in this study can be found in online repositories. The names of the repository/repositories and accession number(s) can be found below: https://github.com/FarmHJ/importance-of-binding-mechanism. The source code and datasets associated to this study is archived on Zenodo at https://doi.org/10.5281/zenodo.7689046.

## References

[B14] Anonymous, ICH E14/S7B Q&A (2022). Questions and answers: Clinical and nonclinical evaluation of QT/QTc interval prolongation and proarrhythmic potential. Available at: https://database.ich.org/sites/default/files/E14-S7B_QAs_Step4_2022_0221.pdf . (Accessed January 24, 2023)

[B15] Anonymous, ICH S7B (2005). S7B the non-clinical evaluation of the potential for delayed ventricular repolarization (QT interval prolongation) by human pharmaceuticals. Available at: https://database.ich.org/sites/default/files/S7B_Guideline.pdf . (Accessed January 24, 2023) 16237859

[B1] Brennan T. Fink M. Rodriguez B. (2009). Multiscale modelling of drug-induced effects on cardiac electrophysiological activity. Eur. J. Pharm. Sci. 36, 62–77. 10.1016/j.ejps.2008.09.013 19061955

[B2] Clayton R. H. Aboelkassem Y. Cantwell C. D. Corrado C. Delhaas T. Huberts W. (2020). An audit of uncertainty in multi-scale cardiac electrophysiology models. Philos. Trans. R. Soc. A 378, 20190335. 10.1098/rsta.2019.0335 PMC7287340 32448070

[B3] Clerx M. Collins P. de Lange E. Volders P. G. A. (2016). Myokit: A simple interface to cardiac cellular electrophysiology. Prog. Biophysics Mol. Biol. 120, 100–114. 10.1016/j.pbiomolbio.2015.12.008 26721671

[B4] Clerx M. Robinson M. Lambert B. Lei C. L. Ghosh S. Mirams G. R. (2019). Probabilistic inference on noisy time series (PINTS). J. Open Res. Softw. 7, 23. 10.5334/jors.252

[B5] Curran M. E. Splawski I. Timothy K. W. Vincen G. M. Green E. D. Keating M. T. (1995). A molecular basis for cardiac arrhythmia: hERG mutations cause long QT syndrome. Cell 80, 795–803. 10.1016/0092-8674(95)90358-5 7889573

[B6] Davies M. R. Mistry H. B. Hussein L. Pollard C. E. Valentin J.-P. Swinton J. (2011). An *in silico* canine cardiac midmyocardial action potential duration model as a tool for early drug safety assessment. Am. J. Physiol. Heart Circ. Physiol. 302, H1466–H1480. 10.1152/ajpheart.00808.2011 22198175

[B7] Di Veroli G. Y. Davies M. R. Zhang H. Abi-Gerges N. Boyett M. R. (2012). High-throughput screening of drug-binding dynamics to hERG improves early drug safety assessment. Am. J. Physiol. Heart Circ. Physiol. 304, H104–H117. 10.1152/ajpheart.00511.2012 23103500

[B8] Di Veroli G. Y. Davies M. R. Zhang H. Abi-Gerges N. Boyett M. R. (2014). hERG inhibitors with similar potency but different binding kinetics do not pose the same proarrhythmic risk: Implications for drug safety assessment. J. Cardiovasc. Electrophysiol. 25, 197–207. 10.1111/jce.12289 24118558

[B9] Dutta S. Chang K. C. Beattie K. A. Sheng J. Tran P. N. Wu W. W. (2017). Optimization of an *in silico* cardiac cell model for proarrhythmia risk assessment. Front. Physiol. 8, 616. 10.3389/fphys.2017.00616 28878692 PMC5572155

[B10] Gomis-Tena J. Brown B. M. Cano J. Trenor B. Yang P.-C. Saiz J. (2020). When does the IC_50_ accurately assess the blocking potency of a drug? J. Chem. Inf. Model. 60, 1779–1790. 10.1021/acs.jcim.9b01085 32105478 PMC7357848

[B11] Hansen N. Müller S. D. Koumoutsakos P. (2003). Reducing the time complexity of the derandomized evolution strategy with covariance matrix adaptation (CMA-ES). Evol. Comput. 11, 1–18. 10.1162/106365603321828970 12804094

[B12] Heist E. K. Ruskin J. N. (2010). Drug-induced arrhythmia. Circulation 122, 1426–1435. 10.1161/CIRCULATIONAHA.109.894725 20921449

[B13] Hindmarsh A. C. Brown P. N. Grant K. E. Lee S. L. Serban R. Shumaker D. E. (2005). Sundials: Suite of nonlinear and differential/algebraic equation solvers. ACM Trans. Math. Softw. 31, 363–396. 10.1145/1089014.1089020

[B16] Kamiya K. Niwa R. Mitcheson J. S. Sanguinetti M. C. (2006). Molecular determinants of hERG channel block. Mol. Pharmacol. 69, 1709–1716. 10.1124/mol.105.020990 16474003

[B17] Kirsch G. E. Trepakova E. S. Brimecombe J. C. Sidach S. S. Erickson H. D. Kochan M. C. (2004). Variability in the measurement of hERG potassium channel inhibition: Effects of temperature and stimulus pattern. J. Pharmacol. Toxicol. Methods 50, 93–101. 10.1016/j.vascn.2004.06.003 15385083

[B18] Lei C. L. Ghosh S. Whittaker D. G. Aboelkassem Y. Beattie K. A. Cantwell C. D. (2020). Considering discrepancy when calibrating a mechanistic electrophysiology model. Philos. Trans. R. Soc. A 378, 20190349. 10.1098/rsta.2019.0349 PMC7287333 32448065

[B19] Li Z. Dutta S. Sheng J. Tran P. N. Wu W. Colatsky T. (2016). A temperature-dependent *in silico* model of the human ether-à-go-go-related (hERG) gene channel. J. Pharmacol. Toxicol. Methods 81, 233–239. 10.1016/j.vascn.2016.05.005 27178106 PMC5042861

[B20] Li Z. Dutta S. Sheng J. Tran P. N. Wu W. Chang K. (2017). Improving the *in silico* assessment of proarrhythmia risk by combining hERG (human Ether-à-go-go-Related Gene) channel-drug binding kinetics and multichannel pharmacology. Circ. Arrhythmia Electrophysiol. 10 10, e004628. 10.1161/CIRCEP.116.004628 28202629

[B21] Milnes J. T. Witchel H. J. Leaney J. L. Leishman D. J. Hancox J. C. (2010). Investigating dynamic protocol-dependence of hERG potassium channel inhibition at 37 degrees C: Cisapride versus dofetilide. J. Pharmacol. Toxicol. Methods 61, 178–191. 10.1016/j.vascn.2010.02.007 20172036

[B22] Mirams G. R. Cui Y. Sher A. Fink M. Cooper J. Heath B. M. (2011). Simulation of multiple ion channel block provides improved early prediction of compounds’ clinical torsadogenic risk. Cardiovasc. Res. 91, 53–61. 10.1093/cvr/cvr044 21300721 PMC3112019

[B23] Mirams G. R. Davies M. R. Cui Y. Kohl P. Noble D. (2012). Application of cardiac electrophysiology simulations to pro-arrhythmic safety testing. Br. J. Pharmacol. 167, 932–945. 10.1111/j.1476-5381.2012.02020.x 22568589 PMC3492977

[B24] Mitcheson J. S. Chen J. Sanguinetti M. C. (2000). Trapping of a methanesulfonanilide by closure of the hERG potassium channel activation gate. J. general Physiol. 115, 229–240. 10.1085/jgp.115.3.229 PMC2217217 10694252

[B25] Mitcheson J. S. (2008). hERG potassium channels and the structural basis of drug-induced arrhythmias. Chem. Res. Toxicol. 21, 1005–1010. 10.1021/tx800035b 18447395

[B26] O’Hara T. Virág L. Varró A. Rudy Y. (2011). Simulation of the undiseased human cardiac ventricular action potential: Model formulation and experimental validation. PLOS Comput. Biol. 7, e1002061. 10.1371/journal.pcbi.1002061 21637795 PMC3102752

[B27] Pearlstein R. A. MacCannell K. A. Erdemli G. Yeola S. Helmlinger G. Hu Q.-Y. (2016). Implications of dynamic occupancy, binding kinetics, and channel gating kinetics for hERG blocker safety assessment and mitigation. Curr. Top. Med. Chem. 16, 1792–1818. 10.2174/1568026616666160315142156 26975508

[B28] Sanguinetti M. C. Jiang C. Curran M. E. Keating M. T. (1995). A mechanistic link between an inherited and an acquired cardiac arrhythmia: HERG encodes the IKr potassium channel. Cell 81, 299–307. 10.1016/0092-8674(95)90340-2 7736582

[B29] Stork D. Timin E. N. Berjukow S. Huber C. Hohaus A. Auer M. (2007). State dependent dissociation of hERG channel inhibitors. Br. J. Pharmacol. 151, 1368–1376. 10.1038/sj.bjp.0707356 17592502 PMC2189824

[B30] Thomas D. Karle C. Kiehn J. (2004). Modulation of hERG potassium channel function by drug action. Ann. Med. 36, 41–46. 10.1080/17431380410032580 15176423

[B31] Tsujimae K. Suzuki S. Murakami S. Kurachi Y. (2007). Frequency-dependent effects of various IKr blockers on cardiac action potential duration in a human atrial model. Am. J. Physiol. Heart Circ. Physiol. 293, H660–H669. 10.1152/ajpheart.01083.2006 17220183

[B32] Vandenberg J. I. Perry M. D. Perrin M. J. Mann S. A. Ke Y. Hill A. P. (2012). hERG K(+) channels: structure, function, and clinical significance. Physiol. Rev. 92, 1393–1478. 10.1152/physrev.00036.2011 22988594

[B33] Whittaker D. G. Clerx M. Lei C. L. Christini D. J. Mirams G. R. (2020). Calibration of ionic and cellular cardiac electrophysiology models. Wiley Interdiscip. Rev. Syst. Biol. Med. 12, e1482. 10.1002/wsbm.1482 32084308 PMC8614115

[B34] Windisch A. Timin E. N. Schwarz T. Stork-Riedler D. Erker T. Ecker G. F. (2011). Trapping and dissociation of propafenone derivatives in hERG channels. Br. J. Pharmacol. 162, 1542–1552. 10.1111/j.1476-5381.2010.01159.x 21175572 PMC3057292

[B35] Yao J. A. Du X. Lu D. Baker R. L. Daharsh E. Atterson P. (2005). Estimation of potency of hERG channel blockers: Impact of voltage protocol and temperature. J. Pharmacol. Toxicol. Methods 52, 146–153. 10.1016/j.vascn.2005.04.008 15936218

[B36] Zhang S. Zhou Z. Gong Q. Makielski J. C. January C. T. (1999). Mechanism of block and identification of the verapamil binding domain to hERG potassium channels. Circ. Res. 84, 989–998. 10.1161/01.res.84.9.989 10325236

