# Supplementary material for: Importance of modelling hERG binding in predicting drug-induced action potential prolongations for drug safety assessment
Source: Front Pharmacol. 2023 Mar 20;14:1110555. doi: 10.3389/fphar.2023.1110555 (PMC10067903; doi:10.3389/fphar.2023.1110555)
Supplement: Supplementary file 1 [file DataSheet1.pdf]

Supplementary Material

Importance of modelling hERG binding in predicting drug-induced action potential prolongations for drug safety assessment

Hui Jia Farm [1], Michael Clerx [2], Fergus Cooper [3], Liudmila Polonchuk [4], Ken Wang [4], David J. Gavaghan [1,3,*] and Chon Lok Lei [5,6,*]

[1]Department of Computer Science, University of Oxford, Oxford, United Kingdom
[2]Centre for Mathematical Medicine and Biology, School of Mathematical Sciences, University of Nottingham, Nottingham, United Kingdom
[3]Doctoral Training Centre, University of Oxford, Oxford, United Kingdom
[4]Roche Pharma Research and Early Development, Pharmaceutical Sciences, Roche Innovation Center Basel, F. Hoffmann-La Roche Ltd., Basel, Switzerland
[5]Institute of Translational Medicine, Faculty of Health Sciences, University of Macau, Macau, China
[6]Department of Biomedical Sciences, Faculty of Health Sciences, University of Macau, Macau, China

Correspondence*: David J. Gavaghan & Chon Lok Lei

1 SYNTHETIC DRUGS

The parameter values of the synthetic drugs are taken from Li et al. (2017). The parameter values for each synthetic drug are given in Table S1.

Table S1. Parameter values of the SD model for all synthetic drugs, taken from Li et al. (2017).

Drug	$K_{\max}$	K_u (ms^{-1})	EC50^n (nM)	N	$V_{\text{half-trap}}$ (mV)
Dofetilide	1.00×10^8	1.79×10^{-5}	5.483×10^8	0.9999	-1.147
Verapamil	4.646×10^4	7.927×10^{-4}	9.184×10^6	1.043	-100
Bepridil	3.735×10^7	1.765×10^{-4}	1.00×10^9	0.9365	-54.93
Terfenadine	9.884×10^3	8.18×10^{-5}	4.138×10^4	0.65	-77.49
Cisapride	9.997	4.161×10^{-4}	4.206×10^1	0.9728	-199.5
Ranolazine	5.584×10^1	1.929×10^{-2}	1.472×10^5	0.95	-94.87
Quinidine	5.770×10^3	1.00×10^{-2}	1.00×10^6	0.8311	-64.87
Bepridil	3.735×10^7	1.765×10^{-4}	1.00×10^9	0.9365	-54.93
Sotalol	2.403×10^3	1.985×10^{-2}	9.619×10^6	0.7516	-55
Chlorpromazine	2.060×10^5	3.866×10^{-2}	5.677×10^7	0.8871	-14.57
Ondansetron	3.354×10^4	2.325×10^{-2}	9.950×10^6	0.8874	-82.11
Diltiazem	2.51×10^2	2.816×10^{-1}	1.00×10^6	0.9485	-90.89
Mexiletine	9.996	9.967×10^{-2}	2.308×10^6	1.304	-86.26

2 PROTOCOLS

The Milnes protocol used in this study is modified from Milnes et al. (2010) by Li et al. (2017). The modified Milnes protocol was repeated with a depolarisation step to $0\,\mathrm{mV}$ from the holding potential of $-80\,\mathrm{mV}$. The $0\,\mathrm{mV}$ step was held for $10\,\mathrm{s}$ before repolarising back to $-80\,\mathrm{mV}$ for $15\,\mathrm{s}$ in between pulses. While the depolarisation step allows the binding of drug compounds to the channel, the $15\,\mathrm{s}$ holding potential in between pulses allows nontrapped drugs to unbind from the channel, thus reducing the inhibition effect on the current.

The *Pneg80*, *P0*, and *P40* protocols from Gomis-Tena et al. (2020) are used to assess the dependency of the SD model and the CS model comparison on the calibration protocol. The Pneg80 protocol was held at a holding potential of $-80\,\mathrm{mV}$, then depolarised to $20\,\mathrm{mV}$ for $0.5\,\mathrm{s}$ before a short pulse of $-50\,\mathrm{mV}$ for $0.2\,\mathrm{s}$. The time period of the protocol was $5.4\,\mathrm{s}$. The P0 and P40 protocols were both held at $-80\,\mathrm{mV}$ holding potential before depolarising to $0\,\mathrm{mV}$ and $40\,\mathrm{mV}$ respectively for $5\,\mathrm{s}$. After that, a short pulse of $-60\,\mathrm{mV}$ was applied for $0.2\,\mathrm{s}$. Both these protocols had pulse length of $5.2\,\mathrm{s}$.

3 APD$_{90}$ VALUES COMPARISON BETWEEN THE AP-SD MODEL AND THE AP-CS MODEL FOR ALL SYNTHETIC DRUGS

The model comparison is repeated for all 12 CiPA training drugs, as listed in Table S1.

3.1 Bepridil

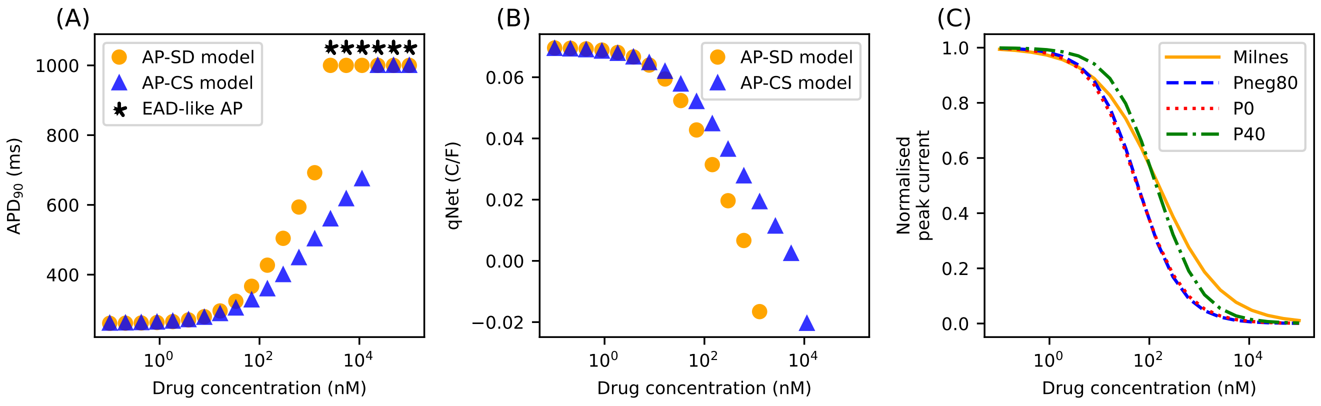

Figure S1. (A) APD$_{90}$s of the AP-SD model and the AP-CS model for bepridil. (B) qNet values of the AP-SD model and the AP-CS model for bepridil. (C) The Hill curves of bepridil from the SD model stimulated by the four protocols: the Milnes, Pneg80, P0, and P40 protocols.

3.2 Terfenadine

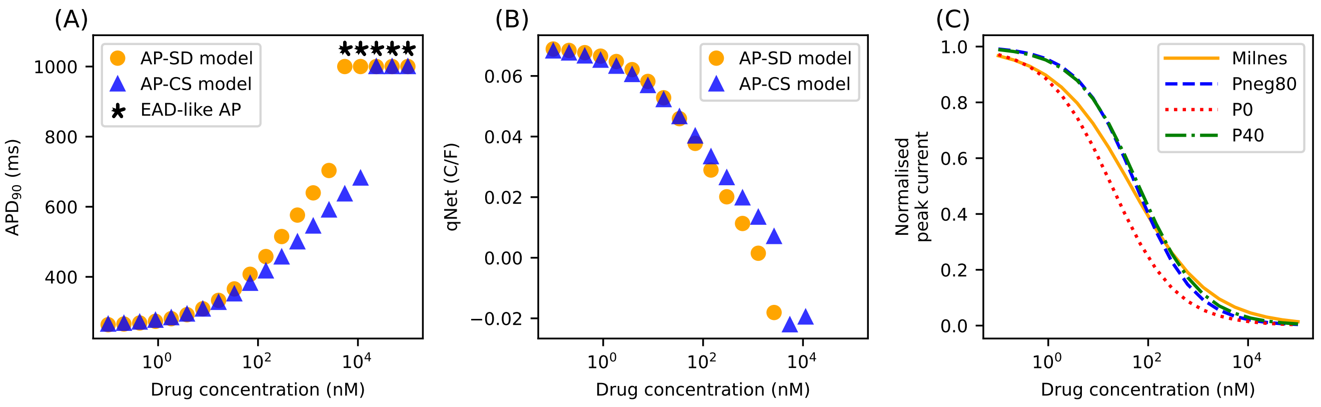

Figure S2. (A) APD_{90}s of the AP-SD model and the AP-CS model for terfenadine. (B) qNet values of the AP-SD model and the AP-CS model for terfenadine. (C) The Hill curves of terfenadine from the SD model stimulated by the four protocols: the Milnes, Pneg80, P0, and P40 protocols.

3.3 Cisapride

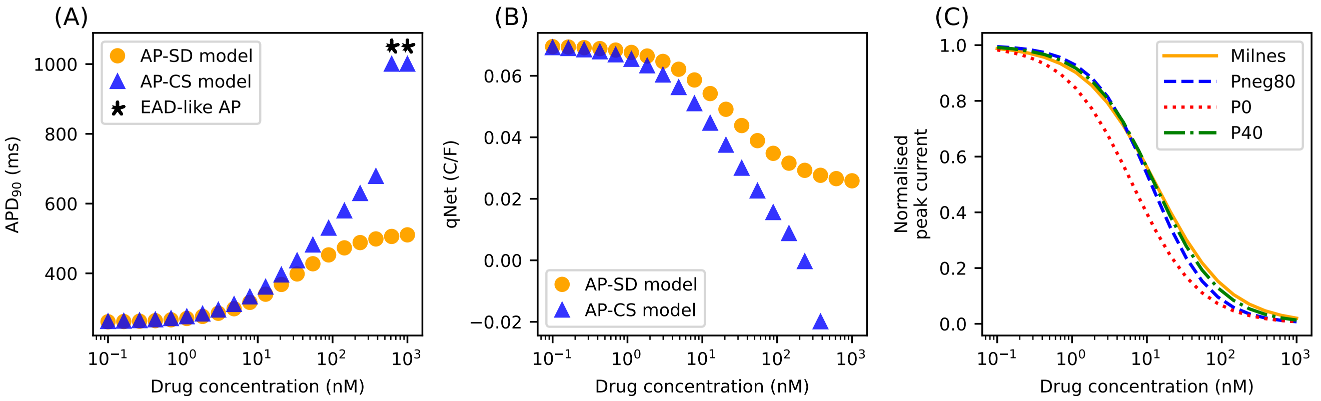

Figure S3. (A) APD_{90}s of the AP-SD model and the AP-CS model for cisapride. (B) qNet values of the AP-SD model and the AP-CS model for cisapride. (C) The Hill curves of cisapride from the SD model stimulated by the four protocols: the Milnes, Pneg80, P0, and P40 protocols.

3.4 Ranolazine

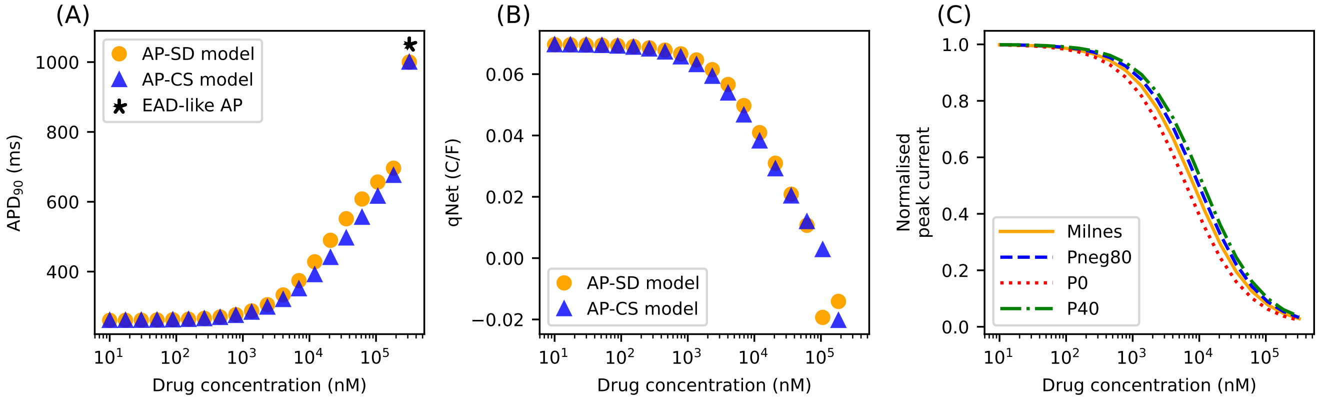

Figure S4. (A) APD_{90}s of the AP-SD model and the AP-CS model for ranolazine. (B) qNet values of the AP-SD model and the AP-CS model for ranolazine. (C) The Hill curves of ranolazine from the SD model stimulated by the four protocols: the Milnes, Pneg80, P0, and P40 protocols.

3.5 Quinidine

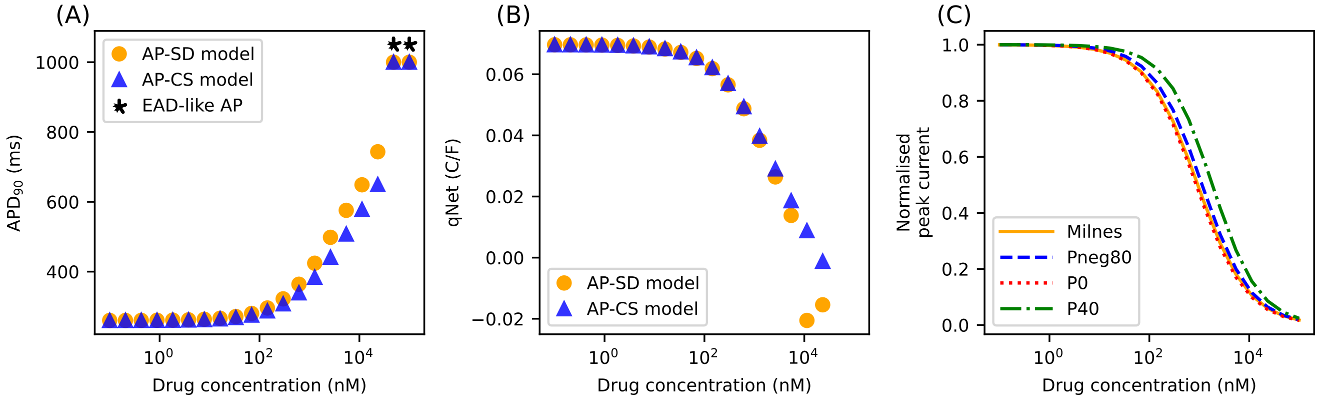

Figure S5. (A) APD_{90}s of the AP-SD model and the AP-CS model for quinidine. (B) The Hill curves of quinidine from the SD model stimulated by the four protocols: the Milnes, Pneg80, P0, and P40 protocols.

3.6 Sotalol

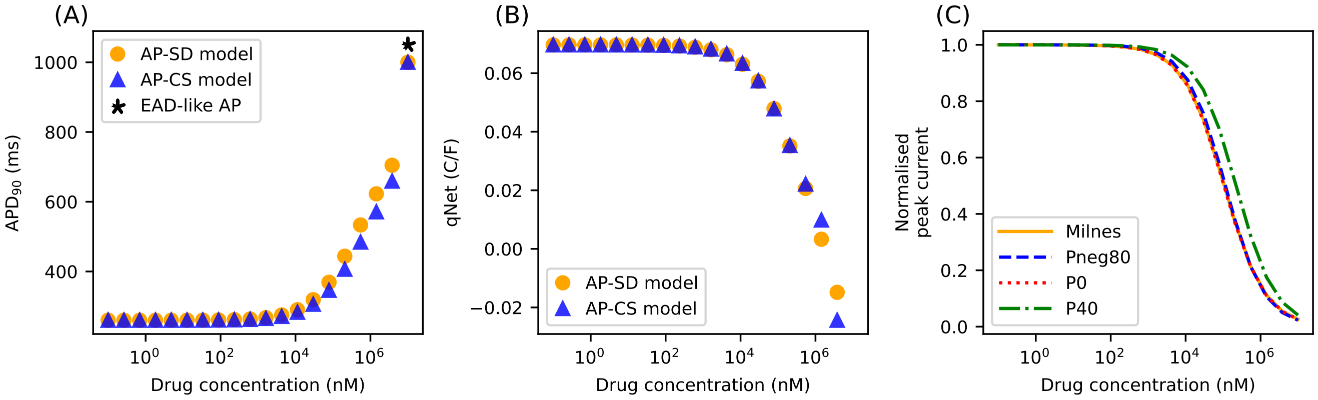

Figure S6. (A) APD_{90}s of the AP-SD model and the AP-CS model for sotalol. (B) qNet values of the AP-SD model and the AP-CS model for sotalol. (C) The Hill curves of sotalol from the SD model stimulated by the four protocols: the Milnes, Pneg80, P0, and P40 protocols.

3.7 Chlorpromazine

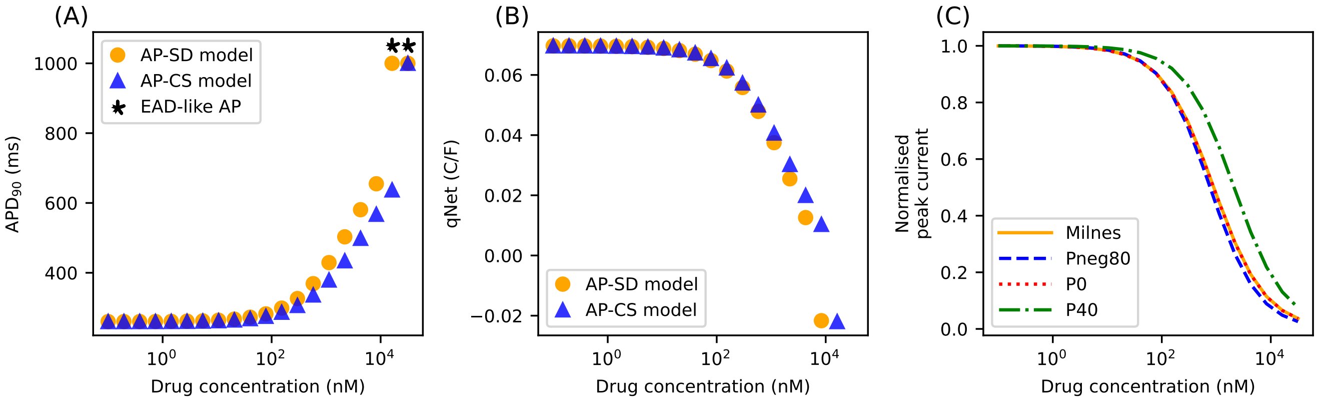

Figure S7. (A) APD_{90}s of the AP-SD model and the AP-CS model for chlorpromazine. (B) qNet values of the AP-SD model and the AP-CS model for chlorpromazine. (C) The Hill curves of chlorpromazine from the SD model stimulated by the four protocols: the Milnes, Pneg80, P0, and P40 protocols.

3.8 Ondansetron

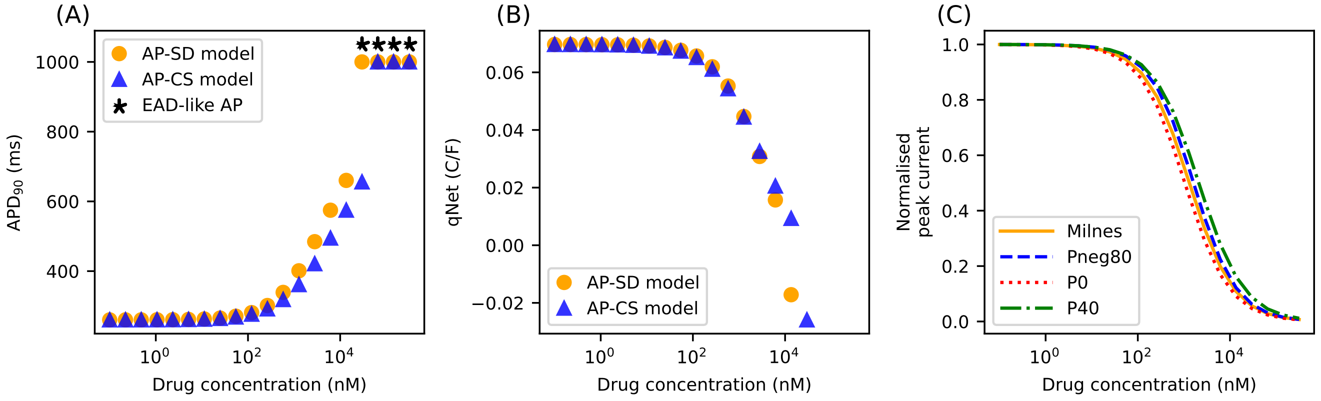

Figure S8. (A) APD_{90}s of the AP-SD model and the AP-CS model for ondansetron. (B) qNet values of the AP-SD model and the AP-CS model for ondansetron. (C) The Hill curves of ondansetron from the SD model stimulated by the four protocols: the Milnes, Pneg80, P0, and P40 protocols.

3.9 Diltiazem

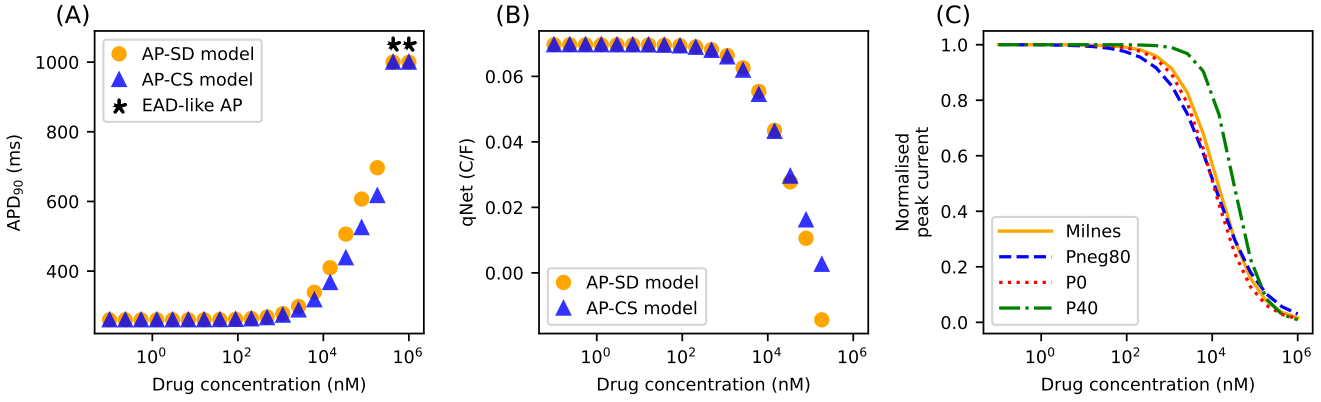

Figure S9. (A) APD_{90}s of the AP-SD model and the AP-CS model for diltiazem. (B) qNet values of the AP-SD model and the AP-CS model for diltiazem. (C) The Hill curves of diltiazem from the SD model stimulated by the four protocols: the Milnes, Pneg80, P0, and P40 protocols.

3.10 Mexiletine

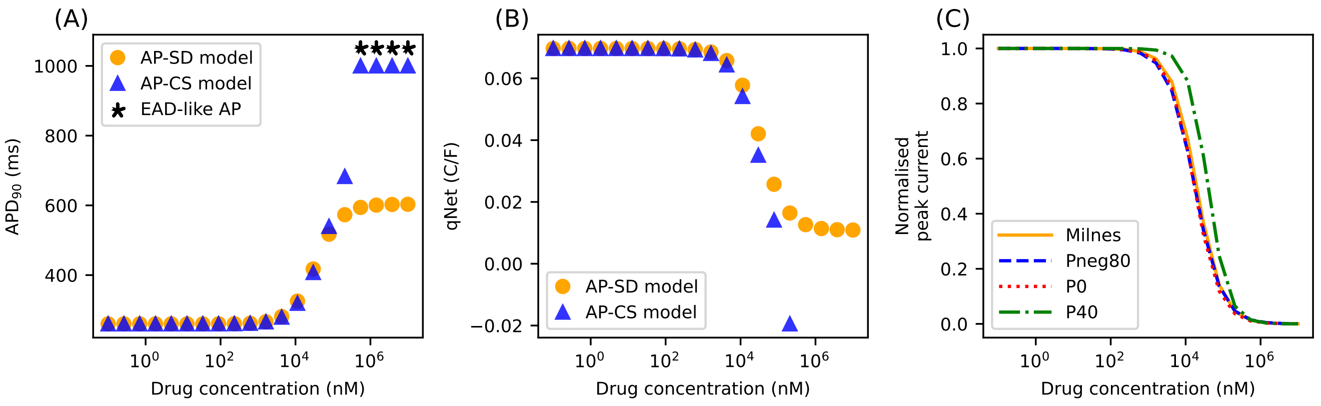

Figure S10. (A) APD_{90}s of the AP-SD model and the AP-CS model for mexiletine. (B) qNet values of the AP-SD model and the AP-CS model for mexiletine. (C) The Hill curves of mexiletine from the SD model stimulated by the four protocols: the Milnes, Pneg80, P0, and P40 protocols.

4 HILL COEFFICIENT APD_{90} DIFFERENCE

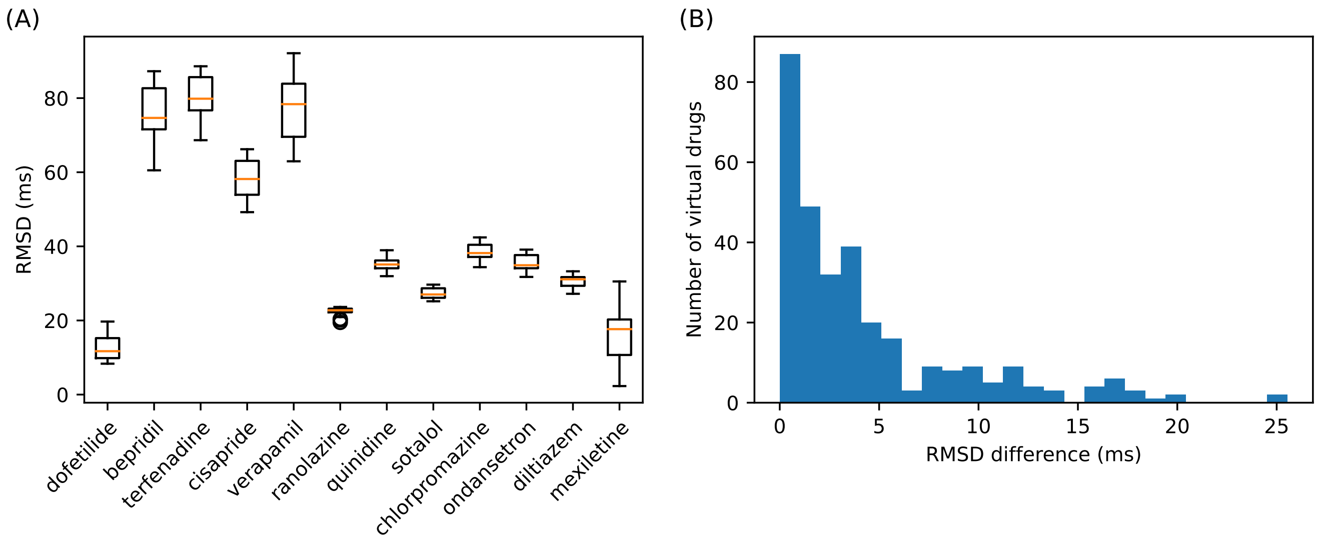

Figure S11. (A) The RMSD of each synthetic drug with the Hill coefficient varied within the minimum and maximum of all synthetic drug's Hill coefficients. (B) A histogram of the difference in RMSD with each synthetic drug's RMSD for all the synthetic drugs.

The sensitivity analysis was performed on the Hill coefficient n for all synthetic drugs. The Hill coefficient was sampled from a range of the minimum and maximum of n of all synthetic drugs. The distribution of the RMSD for each drug is shown Figure S11A. Figure S11B shows the difference in RMSD between the

RMSD of the synthetic drug and the RMSD of the synthetic drug with the Hill coefficient changed for all simulations performed. The RMSD differences had a mean of $4.313 \pm 4.832\,\mathrm{ms}$.

5 SENSITIVITY ANALYSIS: DIFFERENT VIEWING ANGLES

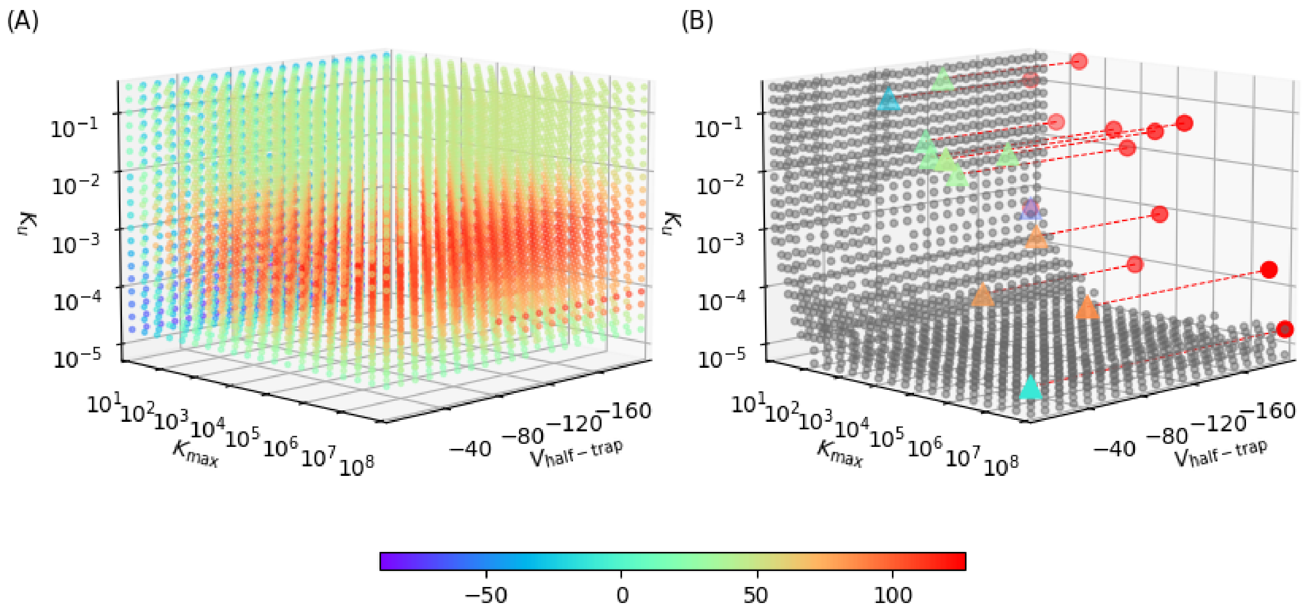

Figure S12. A different viewing angle of Figure 7B. (A) The APD_{90} differences for combinations of $V_{\mathrm{half-trap}}$, K_{max} and K_u parameters. The color of the markers indicate the signed RMSD of each virtual drug in the parameter space. (B) The grey circles are parameter value combination where the signed RMSD is between $-30\,\mathrm{ms}$ and $30\,\mathrm{ms}$. The triangles are the synthetic drugs taken from Li et al. (2017), color coded with their signed RMSD value. These triangles are projected to the K_{max} - K_u plane as red circles for better visualisation.

The parameter values for all 12 synthetic drugs are taken from Li et al. (2017). Of all synthetic drugs, dofetilide, ranolazine, sotalol, and mexiletine showed small APD_{90} differences between the AP-SD model and the AP-CS model. Cisapride showed higher APD_{90} values when it is added to the AP-CS model. The remaining synthetic drugs all caused higher APD_{90} values with the AP-SD model: bepridil, terfenadine, verapamil, quinidine, chlorpromazine, ondansetron, and diltiazem.

6 APD$_{90}$ AT DIFFERENT $V_{\text{half-trap}}$

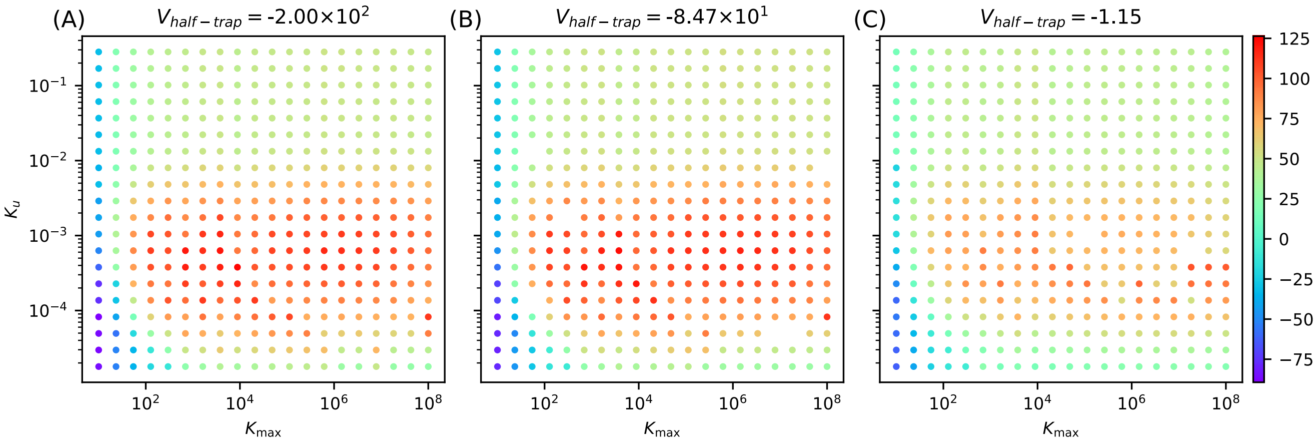

Figure S13. The APD$_{90}$ differences for combinations of K_{max} and K_u at three $V_{\text{half-trap}}$ values. The colour of the markers indicate the signed RMSD of each virtual drug in the parameter space.

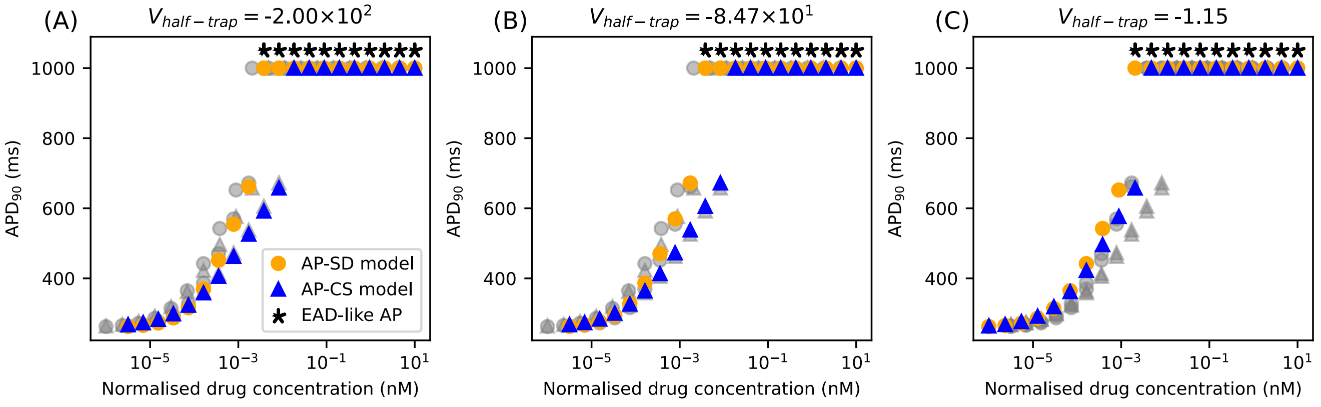

Figure S14. The APD$_{90}$ values for randomly chosen K_{max} and K_u for three $V_{\text{half-trap}}$ values. The grey markers are repeats of the other panels for a better comparison.

In Figure 7, changing $V_{\text{half-trap}}$ does not change the behaviour of the APD$_{90}$ differences. Indeed, the APD$_{90}$ differences for three different $V_{\text{half-trap}}$ with the full range of the K_{max} and K_u axes are similar (Figure S13). However, it does not imply that $V_{\text{half-trap}}$ has no effect on the action potentials. Figure S14 shows the APD$_{90}$ values for the same set of $V_{\text{half-trap}}$ values as in Figure S13, with the same K_{max} and K_u across all panels. The RMSD calculated for the three virtual drugs in Figure S14 are $57.15\,\text{ms}$, $58.88\,\text{ms}$, and $30.29\,\text{ms}$, indicating that the RMSDs are similar (difference between the RMSDs are $< 30\,\text{ms}$) but the APD$_{90}$s are different.

REFERENCES

Gomis-Tena, J., Brown, B. M., Cano, J., Trenor, B., Yang, P.-C., Saiz, J., et al. (2020). When does the IC_{50} accurately assess the blocking potency of a drug? *Journal of Chemical Information and Modeling* 60, 1779–1790. doi:10.1021/acs.jcim.9b01085

Li, Z., Dutta, S., Sheng, J., Tran, P. N., Wu, W., Chang, K., et al. (2017). Improving the in silico assessment of proarrhythmia risk by combining hERG (human Ether-à-go-go-Related Gene) channel-drug binding kinetics and multichannel pharmacology. *Circulation: Arrhythmia and Electrophysiology* 10. doi:10.1161/CIRCEP.116.004628

Milnes, J. T., Witchel, H. J., Leaney, J. L., Leishman, D. J., and Hancox, J. C. (2010). Investigating dynamic protocol-dependence of hERG potassium channel inhibition at 37 °C: Cisapride versus dofetilide. *Journal of Pharmacological and Toxicological Methods* 61, 178–191. doi:10.1016/J.VASCN.2010.02.007
